# Relationship Between Platelet and Urinary 8‐Iso‐PGF2α Levels in Subjects With Different Degrees of NOX2 Regulation

**DOI:** 10.1161/JAHA.113.000198

**Published:** 2013-06-21

**Authors:** Roberto Carnevale, Luigi Iuliano, Cristina Nocella, Simona Bartimoccia, Stefano Trapè, Roberta Russo, Maria Cristina Gentile, Roberto Cangemi, Lorenzo Loffredo, Pasquale Pignatelli, Francesco Violi, IPINET group

**Affiliations:** 1 I Clinica Medica, University of Rome “La Sapienza”, Rome, Italy (R.C., C.N., S.B., S.T., R.R., M.C.G., R.C., L.L., P.P., F.V.); 2 Department of Medico‐Surgical Sciences and Biotechnologies‐Vascular Biology & Mass Spectrometry, University of Rome “La Sapienza”, Rome, Italy (L.I.)

**Keywords:** 8‐iso‐PGF2α, NOX2, oxidative stress, platelets

## Abstract

**Background:**

Urinary 8‐iso‐PGF2α, a marker of oxidative stress, is influenced by the activation of NOX2. It is unclear if platelets 8‐iso‐PGF2α contribute to urinary 8‐iso‐PGF2α.

**Methods and Results:**

In a cross‐sectional study, platelet, urinary, and serum 8‐iso‐PGF2α were determined in subjects with downregulation (X‐linked chronic granulomatous disease [X‐CGD], n=25) and upregulation (type II diabetic patients [T2D], n=121) of NOX2 and 153 controls matched for sex and age. In diabetic patients (n=18), the above variables were repeated before and after 7 days treatment with 100 mg/day aspirin or 100 mg/day aspirin plus 40 mg/day atorvastatin. In vitro study was performed to see the contribution of blood cells to serum 8‐iso‐PGF2α. Compared with controls, X‐CGD patients had lower platelet, serum, and urinary 8‐iso‐PGF2α values; conversely, diabetic patients had higher values of 8‐iso‐PGF2α compared with controls. Urinary 8‐iso‐PGF2α significantly correlated with both platelet and serum 8‐iso‐PGF2α in the 2 cohorts. A parallel increase of platelet, serum, and urinary 8‐iso‐PGF2α by aspirin and a parallel decrease by aspirin plus atorvastatin were detected in the interventional study. In vitro study demonstrated that platelets contribute to 37% of serum 8‐iso‐PGF2α and that only 13% of it is of extravascular origin.

**Conclusions:**

The study suggests that NOX2 contributes to the formation of 8‐iso‐PGF2α in both platelets and urine. The direct correlation between platelet and urinary 8‐iso‐PGF2α suggests that, at least partly, urinary 8‐iso‐PGF2α reflects platelet 8‐iso‐PGF2α production. Analysis of serum 8‐iso‐PGF2α may represent a novel tool to investigate the production of 8‐iso‐PGF2α by blood cells including platelets.

**Clinical Trial Registration:**

URL: ClinicalTrials.gov. Unique Identifier: NCT01250340.

## Introduction

8‐Iso‐PGF2α is a family of eicosanoids deriving from arachidonic acid (AA) oxidation by reactive oxidant species (ROS).^1^ Differently from thromboxane A2, which is chemically unstable aggregating molecules derived from COX1 oxidation of AA,^2^ 8‐iso‐PGF2α are chemically stable compounds with several biologic functions including propagation of platelet activation.^3^ Measurement of 8‐iso‐PGF2α is usually addressed to analyze oxidative stress; in fact, urinary excretion of 8‐iso‐PGF2α has become an established and reliable assay of oxidative stress to be used for clinical purposes in different settings at risk of vascular disease.^4^ However, the cellular contribution to the urinary excretion of 8‐iso‐PGF2α has not been clarified so far. Among the cellular sources of 8‐iso‐PGF2α, platelets play a pivotal role;^5^ therefore, urinary excretion of 8‐iso‐PGF2α could potentially reflect the in vivo release of 8‐iso‐PGF2α on platelet activation. Previous studies have found a direct correlation between urinary 8‐iso‐PGF2α and urinary thromboxane B2 in settings at risk of vascular disease such as diabetes mellitus and hypercholesterolemia, suggesting that urinary 8‐iso‐PGF2α may reflect platelet activation.^5–6^ This issue is potentially relevant, as urinary excretion of 8‐iso‐PGF2α could represent not only a marker of oxidative stress but also a marker of platelet activation potentially usable for clinical purposes. Among the enzymatic pathways implicated in ROS generation and eventually 8‐iso‐PGF2α formation, NOX2, the catalytic core of NADPH oxidase, plays an important role, as suggested by the significant reduction of urinary and platelet 8‐iso‐PGF2α levels in patients with hereditary deficiency of NOX2.^7^ Conversely, subjects with NOX2 upregulation such as diabetic patients have opposite features, as shown by production of platelet 8‐iso‐PGF2α.^8^

Such opposite behavior of urinary 8‐iso‐PGF2α renders these 2 clinical models particularly intriguing to explore the relationship between platelet and urinary 8‐iso‐PGF2α. For this purpose we studied patients with hereditary deficiency of NOX2 or diabetes mellitus and matched controls in whom we measured both urinary excretion of 8‐iso‐PGF2α and platelet production of 8‐iso‐PGF2α. Furthermore, we performed a proof‐of‐concept study to explore the behavior of platelet and urine 8‐iso‐PGF2α in diabetic patients treated with aspirin alone or aspirin plus atorvastatin.

## Methods

### Study Design

To study the relationship between platelet and urinary 8‐iso‐PGF2α in patients with NOX2 downregulation, we conducted a multicenter study in collaboration with the Italian Primary Immunodeficiency Network (IPINET).^9^ Among the 60 patients with CGD registered in the national database, 35 were not included in the study because of the presence of acute infections or critical physical conditions or NADPH oxidase hereditary deficiency unrelated to NOX2 or unwillingness to participate in the study. The remaining 25 patients, who were NOX2 deficient (X‐linked chronic granulomatous disease [X‐CGD]) and willing to participate in the study, were included.

Diagnosis of X‐CGD was performed as previously described.^10^ All X‐CGD patients were under treatment with itraconazole and trimethoprim/sulfamethoxazole.

To study the relationship between platelet and urinary 8‐iso‐PGF2α in patients with NOX2 upregulation, we included consecutive type 2 diabetes mellitus (T2DM) patients attending our metabolic outpatient clinic (n=121). T2DM was diagnosed according to the American Diabetes Association definition.^10^

As a control group, we selected nondiabetic outpatients (n=128) and healthy subjects (n=25) who were matched for age, sex, and history of vascular disease and atherosclerotic risk factors (Table).

**Table 1.** Clinical and Anthropometric Characteristics of Diabetic Patients and Controls

	Diabetic Patients (n=121)*	Controls (n=153)*	*P Value* *
Age, y	67±11	68±13	0.470
Males (%)	59	63	0.341
Smoking habit (%)	20	13	0.301
BMI (kg/m^2^)	27.3±6.0	22.1±3.1	<0.001
Previous coronary artery disease (%)	32	26	0.170
Previous stroke (%)	9	6	0.290
Arterial hypertension (%)	83	81	0.462
Total cholesterol (mg/dL)	185.3±41.0	179.1±43.9	0.258
HDL cholesterol (mg/dL)	51.4±18.0	49.0±14.4	0.508
Triglycerides (mg/dL)	117 (88.0 to 159.0)	124 (88.7 to 168.8)	0.230
Blood glucose (mg/dL)	144.0±42.8	91.5±5.5	<0.001
HbA1c (%)	7.6±1.6	5.1±0.6	<0.001
Statin therapy (%)	57	38	0.001
ACEIs/ARBs (%)	69	64	0.482
Antidiabetic drugs (%)	83	0	<0.001
ASA (100 mg/day)	60	64	0.578
Serum NOX2‐dp (pg/mL)	26.4±11.1	16.0±7.3	<0.001

BMI indicates body mass index; HDL, high‐density lipoprotein; HbA1c, glycated haemoglobin 1Ac; angiotensin‐converting enzyme inhibitors; ARBs, angiotensin receptor blockers; ASA, aspirin; NOX, NADPH oxidase.

* *P*<0.001.

* Diabetic vs controls.

The local ethical committee approved the study protocol (approval no. Prot. 403/09‐Rif. 1621/07.05.09). Each subject enrolled gave informed consent to participate in the study. Clinical characteristics and analysis of urinary 8‐iso‐PGF2α and serum NOX2 have been previously reported.^6^

Exclusion criteria were: (1) liver insufficiency, (2) serious renal disorders (serum creatinine >2.8 mg/dL), (3) cancer, (4) recent history (<3 months) of acute vascular events, (5) treatment with statins or antioxidant vitamins, (6) smokers, (7) clinical diagnosis of type 1 diabetes mellitus (diagnosis of diabetes and insulin use before age 35), (8) active infection, (9) cardiac arrhythmia or congestive heart failure, (10) use of nonsteroidal anti‐inflammatory drugs, and (11) use of antiplatelet drugs such as clopidogrel in the previous 30 days.

Diabetic patients received different antidiabetic treatment: metformin (n=57), subcutaneous insulin (n=25), sulfonylureas (n=14), glinides (n=6), glitazones (n=3), and dipeptidyl peptidase‐4 inhibitor (n=16).

### Laboratory Analyses

Analyses were performed blind. All materials were from Sigma Aldrich unless otherwise specified.

8‐Iso‐PGF2α‐d4 and isoprostane immunoaffinity columns (0.5 mL resin) were purchased from Cayman (Cayman Chemical Europe, Tallin, Estonia). The Altima HP C18 column (100×2.1 mm, 3 μ particles) was from Alltech.

### Blood Sampling

Blood samples were taken after a 12‐hour fast. Between 8:00 and 9:00 am subjects underwent routine biochemical analysis including total cholesterol and glucose. Samples, obtained from patients after supine rest for at least 10 minutes, were taken in tubes with 3.8% sodium citrate and centrifuged at 300*g* for 10 minutes to obtain supernatant. Blood samples without anticoagulant were kept for 60 minutes at 37°C or at room temperature and centrifuged 10 minutes at 300*g*. Plasma and serum samples were immediately stored at −80°C until use with the antioxidant butylated hydroxytoluene at final concentration of 20 μmol/L.

### Platelet Preparation

To obtain platelet‐rich plasma (PRP), samples were centrifuged for 15 minutes at 180*g*. To avoid leukocyte contamination, only the top 75% of the PRP was collected according to Pignatelli et al.^3^ Platelet pellets was obtained by double centrifugation (5 minutes, 300*g*) of PRP. Acid/citrate/dextrose (ACD; 1:7 *v*/*v*) was added to avoid platelet activation during processing; samples were suspended in HEPES buffer in the presence of 0.1% albumin, pH 7.354 (2×10^5^ platelets/mL, unless otherwise noted), and stimulated with or without 0.5 mM AA in the presence or absence of sNOX2dp‐tat (10 μM), an inhibitor of NOX2 activation. Cells were separated from the supernatant by centrifugation (5 minutes, 300*g*) and stored until analysis. Values of 8‐iso‐PGF2α, expressed as picomoles per liter, were obtained by subtracting the concentration of 8‐iso‐PGF2α obtained from acellular PBS incubated for 30 minutes at 37°C with 0.5 mM AA.

### Human Polymorphonuclear Leukocytes Preparation

Polymorphonuclear leukocytes (1×10^6^ cells/mL) were isolated from freshly taken EDTA blood from healthy volunteers (n=5) by dextran‐enhanced sedimentation of red blood cells, Ficoll‐Histopaque density centrifugation, lysis of remaining erythrocytes with distilled water, and washing of cells with Hank's balanced salt solution (HBSS) in the absence of any divalent cations. Finally, the cell pellet was suspended in 1 mL of HBSS and stimulated with or without 10 μM of phorbol 12‐myristate 13‐acetate in the presence or absence of sNOX2dp‐tat (10 μM), an inhibitor of NOX2 activation. Cells were separated from the supernatant by centrifugation (5 minutes, 300*g*) and stored until analysis.

### Lymphocyte/Monocyte Preparation

Blood samples were collected in heparinized tubes (10 IU/mL). Lymphocytes/monocytes (1×10^6^ cells/mL) were isolated after centrifugation of the blood from healthy volunteers (n=5) with a polysucrose‐sodium diatrizoate solution, 1.077 g/mL density and 280 mOsm osmolarity (Lymphoprep; Nycomed, Oslo, Norway), at 800*g* at 20°C. The lymphocyte/monocyte cell layer was collected, and the cells were thus washed 2 times in a solution of cold phosphate‐buffered saline (pH 7.2) supplemented with 1% fetal calf serum and 2 mmol/L EDTA (Sigma‐Aldrich, Milano, Italy). The cell suspension was stimulated with or without lipopolysaccharide (100 ng/mL) in the presence or absence of sNOX2dp‐tat (10 μM), an inhibitor of NOX2 activation. Cells were separated from the supernatant by centrifugation (5 minutes, 300*g*) and stored until analysis.

### 8‐Iso‐PGF2α Level Assays in Liquid Chromatography/Mass Spectrometry

Urinary samples were collected and immediately stored with the antioxidant butylated hydroxytoluene at final concentration of 20 μmol/L.

8‐iso‐PGF2α was analyzed by isotope dilution liquid chromatography/mass spectrometry as reported by Sircar et al.^11^ Samples were added with tetra‐deuterated iPF2α‐III and purified on immunoaffinity column according to the manufacturer's instructions. Before immunoaffinity, purification serum samples were subjected to alkaline hydrolysis for 40 minutes at 60°C, and at the end of incubation, pH was adjusted to 7.4. Column eluate was evaporated under a stream of N_2_ and redissolved in a 100‐μL mobile phase consisting of 20% acetonitrile and 0.1% acetic acid I water. Twenty microliters of sample was chromatographed on an Altima HP C18 column (100×2.1 mm, 3μ particles; Alltech) by an Agilent quaternary pump system using a linear gradient of mobile phase A to mobile phase B (45% acetonitrile, 0.1% acetic acid) for 25 minutes at a flow rate of 200 μL/min. Mass spectrometry was performed on an HCT plus ion‐trap instrument (Bruker Daltonics, Bremen, Germany) operating with an electron spray ion source. Single‐ion monitoring acquisition included 353.2 and 357.2 *m*/*z* ions for 8‐iso‐PGF2α and 8‐iso‐PGF2α‐d4, respectively. Concentration was calculated using an isotope ratio standard curve.

Urinary 8‐iso‐PGF2α concentration was corrected for recovery and creatinine excretion, and values are expressed as picograms per milligram of creatinine. Serum values are expressed as picograms per milliliter and platelets values are expressed as pg/mL×10^8^.

### Interventional Study

Diabetic patients (n=18) were treated for 7 days with 100 mg/day aspirin and atorvastatin 10 mg/day plus aspirin for an additional 7 days. There was an interval of 10 days between the 2 phases of the study. Adherence to aspirin or atorvastatin treatment was assessed by the pill count method. Blood samples were collected at baseline and after 7 days of treatment. The study was registered in August 2010 at ClinicalTrials.gov (Identifier: NCT01250340). At each scheduled time, platelet, serum, and urinary 8‐iso‐PGF2α were determined.

### Statistical Analysis

## Sample size determination

As above reported, for the cross‐sectional study we recruited all the subjects who respected the inclusion/exclusion criteria. The number of patients and controls was computed with respect to a 2‐tailed Student *t* test for independent groups, considering (1) a difference in serum 8‐iso‐PGF2α to be detected between diabetic patients and controls, |δ|≥150 pmol/L; (2) standard deviations homogeneous between groups, SD=200 pmol/L; and (3) type I error probability α=0.05 and power 1−β=0.90. This resulted in n=39 per group, which was increased to 50.

As regard the interventional cross‐over study, we computed the minimum sample size with respect to a 2‐tailed 1‐sample Student *t* test, considering (1) a difference for serum 8‐iso‐PGF2α variation to be detected between baseline and after aspirin+atorvastatin treatment, |δ|≥150 pmol/L; (2) standard deviation of the paired differences, SD=180 pmol/L; and (3) type I error probability α=0.05 and power 1−β=0.90. This resulted in n=18.

## Statistical methods

Categorical variables are reported as counts (percentages) and continuous variables as means±SDs unless otherwise indicated. Independence of categorical variables was tested by the *χ*^2^ test. Comparisons between patients and controls were carried out by the Student *t* test and were replicated as appropriate with nonparametric tests (Kolmogorov–Smirnov [*z*] test in case of nonhomogeneous variances, as verified by Levene's test).

The crossover study data were analyzed for the assessment of treatment and period effects by performing an ANOVA test for repeated measures. Pairwise comparisons were corrected by the Bonferroni test.

Bivariate analysis was performed with the Spearman linear regression test.

## Results

### 8‐Iso‐PGF2α Levels in Patients With NOX2 Downregulation

**Figure 1.**
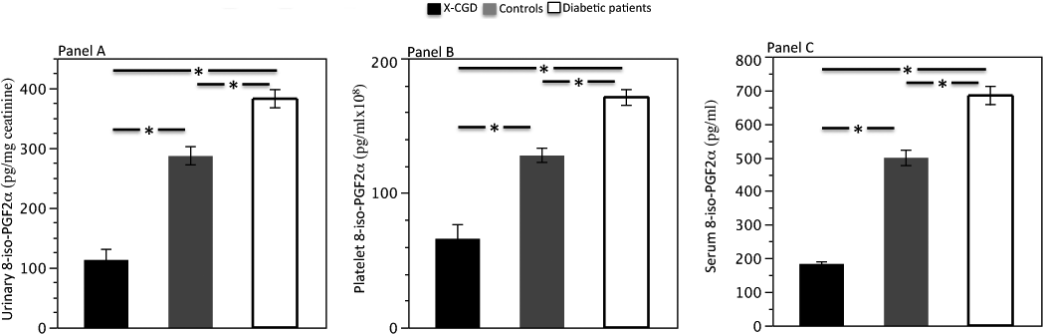
Urinary (A), platelet (B), and serum (C) 8‐iso‐PGF2α levels in X‐CGD patients (n=25), controls (n=153), and diabetic patients (n=121); *P*<0.001. The error bar represents the standard error. X‐CGD indicates X‐linked chronic granulomatous disease; PGF2a, 8‐iso‐Prostaglandin F2‐alpha.

As previously reported, no differences in clinical characteristic were detected in patients with NOX2 hereditary deficiency and controls, whereas sNOX2‐dp was significantly lower in patients with NOX2 hereditary deficiency than in controls.^7^ 8‐Iso‐PGF2α levels varied widely according to the sample studied. Compared with controls, patients with NOX2 hereditary deficiency had lower production of platelet, urinary, and serum 8‐iso‐PGF2α (Figure 1). Whatever the method used, patients with NOX2 hereditary deficiency showed ≥50% 8‐iso‐PGF2α formation reduction compared with controls. In the whole population serum, urinary and platelet 8‐iso‐PGF2α levels significantly correlated each other (*R*=0.550, *P*<0.001, for serum versus urinary 8‐iso‐PGF2α levels; *R*=0.583, *P*<0.001, for platelet versus urinary 8‐iso‐PGF2α levels; and *R*=0504, *P*<0.001, for serum versus platelet 8‐iso‐PGF2α levels).

### 8‐Iso‐PGF2α Levels in Patients With NOX2 Upregulation

No differences in clinical characteristics were detected in diabetic patients compared with controls with the exception of glycemic control, body mass index, and blood glucose (Table). Serum sNOX2‐dp was significantly higher in diabetic patients compared with controls (Table).

In the whole population the amount of 8‐iso‐PGF2α greatly varied according to the method used, with the largest quantity of 8‐iso‐PGF2α found in serum compared with urine and platelets. Whatever the method used, a different 8‐iso‐PGF2α formation was observed between diabetic patients and controls. Thus, urinary, serum, and platelet 8‐iso‐PGF2α levels were significantly higher in diabetic patients compared with controls (Figure 1). In the whole population, serum, urinary, and platelet 8‐iso‐PGF2α levels significantly correlated with each other (*R*=0.475, *P*<0.001, for serum versus urinary 8‐iso‐PGF2α levels; *R*=0.602, *P*<0.001, for platelet versus urinary 8‐iso‐PGF2α levels; and *R*=0.505, *P*<0.001, for serum versus platelet 8‐iso‐PGF2α levels).

**Figure 2.**
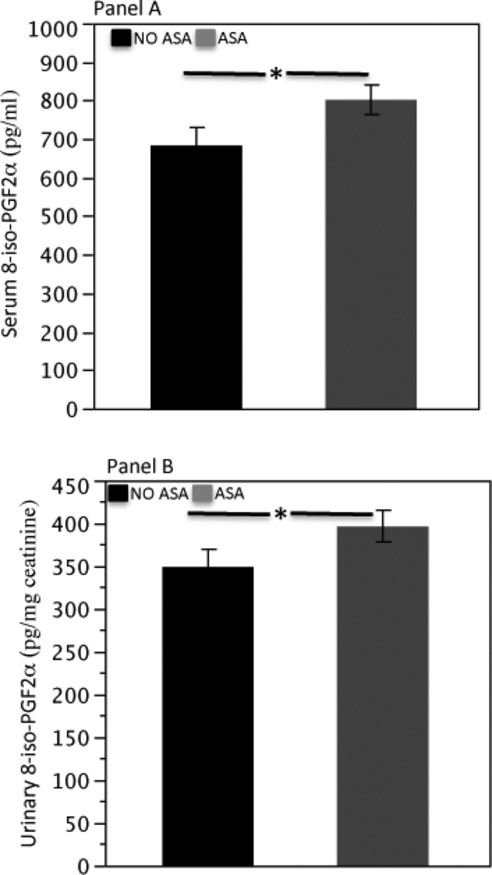
Serum (A) and urinary (B) 8‐iso‐PGF2α formation in diabetic patients treated or not treated with aspirin (100 mg/day). The error bar represented the standard error. ASA indicates aspirin; PGF2a, 8‐iso‐Prostaglandin F2‐alpha.

As a previous study showed an increase in platelet 8‐iso‐PGF2α in aspirin‐treated diabetic patients,^9^ we investigated if similar changes were detected in serum and urine. As shown in Figure 2, aspirin‐treated diabetic patients had significantly higher serum and urine 8‐iso‐PGF2α levels compared with untreated patients.

### Overall Analysis of NOX2 and 8‐Iso‐PGF2α Levels in the Entire Population

**Figure 3.**
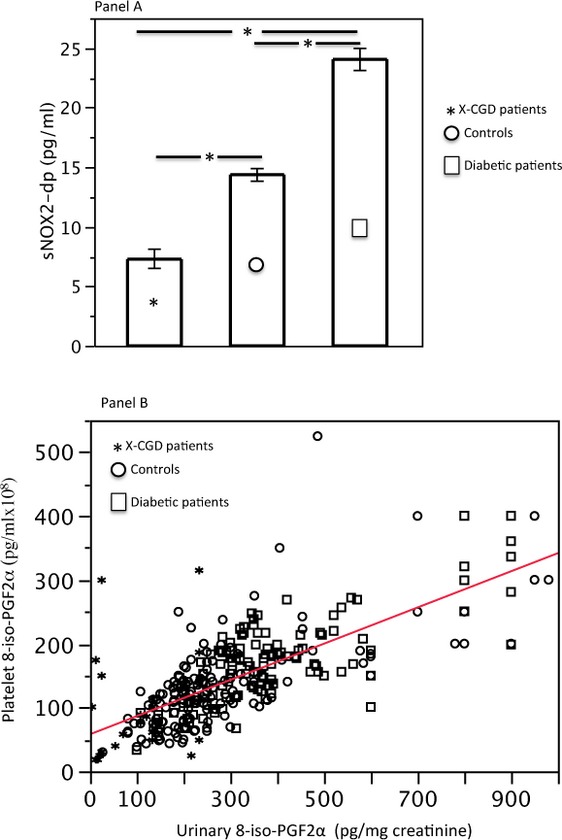
A, Serum sNOX‐2‐dp levels in X‐CGD patients, controls, and diabetic patients (the error bar represented the standard error). B, Urinary (on the *x* axis) and platelet (on the *y* axis) 8‐iso‐PGF2α levels in both hereditary deficiency of NOX2, controls, and diabetic patients (highlighted with different symbols; data are represented as a scatter plot). X‐CGD indicates X‐linked chronic granulomatous disease; NOX, NADPH oxidase; sNOX‐2‐dp, soluble NADPH oxidase 2‐derived peptide.

A progressive increase in NOX2 activity was detected from X‐CGD to diabetic patients (Figure 3A). A significant association between platelet and urinary 8‐iso‐PGF2α levels was detected in the overall population (X‐CGD, controls, and diabetic patients; *R*=0.518, *P*<0.001; Figure 3B).

### Interventional Study

**Figure 4.**
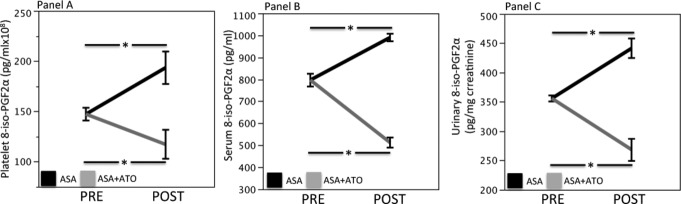
Platelet (A), serum (B), and urinary (C) 8‐iso‐PGF2α formation in diabetic patients at baseline and after 7 days of aspirin (100 mg/day) or aspirin (100 mg/day) plus atorvastatin (10 mg/day) treatment. The error bar represented the standard error. ASA indicates aspirin; ATO, atorvastatin.

As previously reported,^8^ diabetic patients showed platelet 8‐iso‐PGF2α overexpression after 7 days of aspirin treatment; such an increase was parallel to 8‐iso‐PGF2α overexpression in serum and urine (Figure 4B and 4C). The combination of aspirin with atorvastatin resulted in a significant decrease in platelet 8‐iso‐PGF2α levels (Figure 4A); similar behavior was detected by 8‐iso‐PGF2α measured in serum and urine (Figure 4B and 4C). Changes in urinary 8‐iso‐PGF2α levels were significantly correlated with changes in platelets (*R*=0.500, *P*<0.001) and serum 8‐iso‐PGF2α levels (*R*=0.410, *P*<0.001).

### In Vitro Study

**Figure 5.**
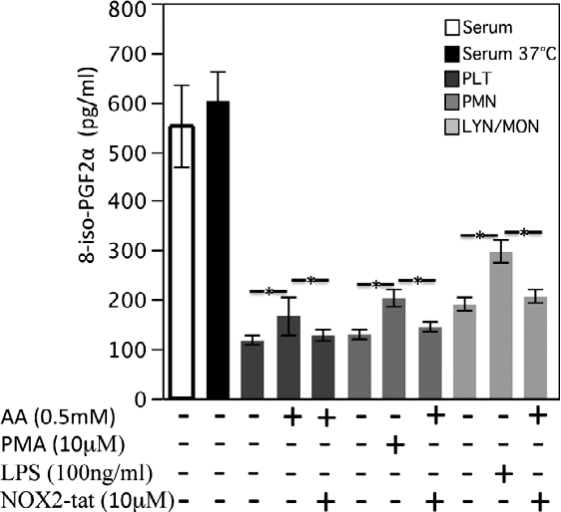
8‐Iso‐PGF2α levels in serum kept for 60 minutes at 37°C (n=5) or at room temperature (n=5) in platelets stimulated with AA in the presence or absence of NOX2‐tat inhibitor (n=5), in PMNs stimulated with PMA in the presence or absence of NOX2‐tat inhibitor (NADPH oxidase inhibitor; n=5), and in LYN/MON stimulated with LPS in the presence or absence of NOX2‐tat inhibitor (n=5); *P<0.001. The error bar represented the standard error. AA indicates arachidonic acid; PMNs, polymorphonuclear leukocytes; PMA, phorbol 12‐myristate 13‐acetate; PGF2a, 8‐iso‐Prostaglandin F2‐alpha; NOX, NADPH oxidase; LYM/MON, lymphocytes/monocyte; LPS, lipopolysaccharide.

As serum 8‐epi‐PGF_2_ significantly correlated with platelet 8‐iso‐PGF2α, we speculated that 8‐iso‐PGF2α levels detected in serum may reflect platelet 8‐iso‐PGF2α production. To address this point, we measured 8‐iso‐PGF2α levels from blood cells including platelets, leukocytes, and lymphocytes and compared the production of each cell line with serum 8‐iso‐PGF2α levels. Thus, a parallel analysis of serum and cell production of 8‐iso‐PGF2α levels was performed in blood samples taken from 5 healthy subjects (3 men and 2 women, aged 52.2±2.1 years). 8‐Iso‐PGF2α levels varied according to the cell lines, with the highest values detected by agonist‐stimulated lymphocytes (296.93±75.43 pg/mL). The sum of 8‐iso‐PGF2α produced by the 3 cell lines was 434.90 pg/mL and corresponded to 80% of the serum 8‐iso‐PGF2α levels. The amount of agonist‐induced platelet 8‐iso‐PGF2α formation was 166.16±106.78 pmol/L and corresponded to 30.1% of serum 8‐epi‐PGF2α. For each cell line, 8‐iso‐PGF2α level was significantly inhibited by cell incubation with the specific NADPH oxidase inhibitor NOX2‐tat (Figure 5). Serum 8‐iso‐PGF2α level was not influenced by temperature, as the amount detected in the supernatant of serum left at room temperature was similar to that incubated for 60 minutes at 37°C (Figure 5).

## Discussion

The results of the present study demonstrate that in patients with up‐ and downregulation of NOX2, platelet and urinary 8‐iso‐PGF2α were up‐ and down‐expressed, respectively, suggesting that NOX2 is implicated in the formation of 8‐iso‐PGF2α detected in platelets and urine. The correlation between platelet and urinary 8‐iso‐PGF2α suggests also that urinary 8‐iso‐PGF2α may reflect, in part, platelet 8‐iso‐PGF2α production. This hypothesis was reinforced by an interventional study showing that up‐ and downregulation of platelet 8‐iso‐PGF2α levels were associated with parallel behavior of 8‐iso‐PGF2α levels in urine.

We have previously demonstrated that NOX2 plays a crucial role in 8‐iso‐PGF2α formation. In fact, in patients with hereditary deficiency of NOX2 and in patients with upregulation of NOX2 such as those with diabetes, platelet production of 8‐iso‐PGF2α was reduced and increased, respectively.^3,8^ Such interplay was reinforced by an in vitro study in which platelet incubation with a specific inhibitor of NOX2 was associated with impaired production of 8‐iso‐PGF2α.^3^ We speculated that these 2 settings were unique in investigating if urinary 8‐iso‐PGF2α could reflect platelet production of 8‐iso‐PGF2α. This issue has potential clinical implications because the increase in urinary 8‐iso‐PGF2α levels has been interpreted essentially as a specific marker of oxidative stress so far.^4^ The results of the cross‐sectional study suggest that urinary 8‐iso‐PGF2α levels could reflect, in part, platelet production of 8‐iso‐PGF2α. Thus, in patients with lowered or increased urinary 8‐iso‐PGF2α levels, as observed in patients with NOX2 hereditary deficiency and diabetes, respectively, parallel platelet 8‐iso‐PGF2α behavior was detected. Also, in both cohorts the 2 variables were significantly correlated. This finding may help to explain previous findings showing a significant correlation between urinary excretion of 8‐iso‐PGF2α and 11‐dehydro‐thromboxane B2 and suggesting that changes in both variables reflect in vivo platelet activation.^5–6^ However, we cannot exclude that in diabetes the increase may stem from activation of other cells such as leukocytes and monocytes, which are also implicated in the activation of NOX2.^12^

The results of the cross‐sectional study in subjects with up‐ and downregulation of NOX2 were corroborated by the interventional study with aspirin and aspirin plus atorvastatin in diabetic patients. We have recently shown that in patients with diabetes mellitus, aspirin shifts arachidonic acid to nonenzymatic oxidation^8^ via NOX2 activation, an effect that ultimately leads to platelet 8‐iso‐PGF2α overexpression.^8^ In the present study we found that after aspirin intake, 8‐iso‐PGF2α overexpression was detected not only in platelets but also in urine and serum. Administration to aspirin‐treated diabetic patients of atorvastatin, a drug that lowers NOX2 activation,^12–13^ counteracted such a phenomenon by inhibiting platelet 8‐iso‐PGF2α in platelets, serum, and urine. The changes in platelet 8‐iso‐PGF2α levels observed in the 2 phases of therapy were parallel and significantly correlated with changes in urinary 8‐iso‐PGF2α levels, reinforcing the hypothesis that platelet production of 8‐iso‐PGF2α influences urinary excretion of this eicosanoid family.

Another novelty of the present study is the measurement of 8‐iso‐PGF2α levels in serum and the analysis of the relative contribution of platelets. Here we report that 8‐iso‐PGF2α levels in serum are double that of the amount detected in urine. Globally considered, blood cell activation contributes to 80% of total serum 8‐iso‐PGF2α, and platelet activation accounts for about 30% of serum 8‐iso‐PGF2α. This finding may explain the significant correlation observed between platelet and serum 8‐iso‐PGF2α levels in the cross‐sectional as well as the interventional study. Hence, the correlation between serum 8‐iso‐PGF2α level and urinary 8‐iso‐PGF2α level corroborates the hypothesis that platelet 8‐iso‐PGF2α levels partly contribute to the urinary excretion of this eicosanoid. It is also of interest that similar values of serum 8‐iso‐PGF2α levels were detected in samples left at room temperature or incubated for 30 minutes at 37°C. Thus, serum storage at −80°C may be a simple approach to measure 8‐iso‐PGF2α production by blood cells, including platelets.

The study has potential limitations. The hypothesis of a role of platelet 8‐iso‐PGF2α formation as a contributor to urinary 8‐iso‐PGF2α levels is essentially based on correlations analysis between platelets and urinary 8‐iso‐PGF2α levels. It is noteworthy, however, that in patients with NOX2 hereditary deficiency and impaired platelet 8‐iso‐PGF2α formation, urinary 8‐iso‐PGF2α levels were coincidentally reduced. Furthermore, the interventional study with atorvastatin demonstrated that reduction of platelet 8‐iso‐PGF2α was significantly associated with urinary 8‐iso‐PGF2α level lowering. Of course, as shown by in vitro experiments, other blood cells producing 8‐iso‐PGF2α can contribute to urinary 8‐iso‐PGF2α levels. Hence, up‐ or down‐expression of serum and urine cannot be solely interpreted as related to platelet activation but also to activation of other blood cells. In this context the use of serum 8‐iso‐PGF2α levels for clinical purposes is still premature, as a prospective study needs to be done to support its validity.

In conclusion, our study provides evidence that NOX2 activity is implicated in 8‐iso‐PGF2α formation detected in platelets and urine. The correlation between platelet and urine 8‐iso‐PGF2α levels suggests that 8‐iso‐PGF2α levels in urine might in part reflect platelet activation. Analysis of 8‐iso‐PGF2α levels in serum may represent a simple approach to investigating 8‐iso‐PGF2α production by blood cells, including platelets, in patients at risk of cardiovascular disease.
